# Structure, Health Benefits, Mechanisms, and Gut Microbiota of *Dendrobium officinale* Polysaccharides: A Review

**DOI:** 10.3390/nu15234901

**Published:** 2023-11-23

**Authors:** Weijie Wu, Ziqi Zhao, Zhaoer Zhao, Dandan Zhang, Qianyi Zhang, Jiayu Zhang, Zhengyi Fang, Yiling Bai, Xiaohui Guo

**Affiliations:** 1 College of Food Science and Nutritional Engineering, China Agricultural University, Beijing 100083, China; weijiewu@cau.edu.cn (W.W.); zzq2018@cau.edu.cn (Z.Z.); zhaoer2019@gmail.com (Z.Z.); haleyz@cau.edu.cn (D.Z.); zhangqy@cau.edu.cn (Q.Z.); baiyiling@cau.edu.cn (Y.B.); 2 School of Public Health, Chengdu University of Traditional Chinese Medicine, Chengdu 611137, China; jiayuzz1998@gmail.com; 3 School of Clinical Medicine, Chengdu University of Traditional Chinese Medicine, Chengdu 611137, China; sync_1900@stu.cdutcm.edu.cn

**Keywords:** *Dendrobium offcinale* polysaccharides, gut microbiota, metabolism regulation, inflammation modulation, immunity moderation, cancer intervention

## Abstract

*Dendrobium officinale* polysaccharides (DOPs) are important active polysaccharides found in *Dendrobium officinale*, which is commonly used as a conventional food or herbal medicine and is well known in China. DOPs can influence the composition of the gut microbiota and the degradation capacity of these symbiotic bacteria, which in turn may determine the efficacy of dietary interventions. However, the necessary analysis of the relationship between DOPs and the gut microbiota is lacking. In this review, we summarize the extraction, structure, health benefits, and related mechanisms of DOPs, construct the DOPs-host axis, and propose that DOPs are potential prebiotics, mainly composed of 1,4-β-D-mannose, 1,4-β-D-glucose, and O-acetate groups, which induce an increase in the abundance of gut microbiota such as *Lactobacillus*, *Bifidobacterium*, *Akkermansia*, *Bacteroides*, and *Prevotella*. In addition, we found that when exposed to DOPs with different structural properties, the gut microbiota may exhibit different diversity and composition and provide health benefits, such as metabolism regulations, inflammation modulation, immunity moderation, and cancer intervention. This may contribute to facilitating the development of functional foods and health products to improve human health.

## 1. Introduction

*Dendrobium officinale*, a member of the Orchidaceae family, is mainly distributed in Anhui and Zhejiang provinces in China, Kyushu Island in Japan, and the state of Missouri in the United States [1]. In China, it is often used as a conventional food or herbal medicine and is known for its non-toxic and harmless properties [2]. *Dendrobium officinale* is considered the most advanced herbal medicine in China and is cultivated in many provinces in China [3]. DOPs are important bioactive constituents of *Dendrobium officinale*; water-soluble, non-starchy, active polysaccharides extracted from *Dendrobium officinale* account for about 20% [4]. They consist mainly of 1,4-β-D-mannose, 1,4-β-D-glucose, and O-acetate groups, and both in vivo and in vitro experiments have shown that DOPs are resistant to digestion and are mainly metabolized by the gut microbiota in the large intestine, with subsequent microbial metabolism in the intestine yielding oligosaccharides and short-chain fatty acids (SCFAs) that influence gut microbiota diversity and compositional structure and promote human health. Therefore, DOPs exhibits potential to act as prebiotics [5,6,7,8,9]. The nutritional value of DOPs has been confirmed as they inhibit the abundance of harmful bacteria in the gut, such as *Escherichia* and *Staphylococcus* [10], while increasing the abundance of beneficial bacteria, such as *Bifidobacterium* [11]. Evidence from human and animal studies suggests that consumption of DOPs may alleviate metabolic disorders such as obesity [12,13] and type 2 diabetes [14,15] and may influence inflammation such as the deterioration of lung function in chronic obstructive pulmonary disease [16,17], immunomodulation [18], and cancer intervention such as gastric precancerous lesions [19].

In recent years, several scientists have conducted comprehensive studies on DOPs. For example, Chen et al. [20] provided an overview of the research progress on the isolation, structural properties, and biological activities of DOPs. In addition, He et al. [21] provided a systematic review on the extraction, isolation, purification, structural properties, biological activities, mechanism of action, and structure–activity relationships of DOPs. However, the necessary analysis of the essential relationship between DOPs as potential prebiotics and the gut microbiota is lacking. In this paper, the extraction, structure, biological activities, mechanism of action, and structure–activity relationships of DOPs are re-examined, focusing on exploring the relationship between DOPs and gut microbiota to provide guidance for the further development of DOPs.

## 2. Extraction and Structure of Polysaccharides from *Dendrobium officinale*

In order to obtain bioactive polysaccharides from *Dendrobium officinale*, the methods of extraction, purification, and analysis have been continuously optimized for both research and industrial production. Fresh *Dendrobium officinale* is washed, dried, and ground to a powder at low temperatures. The crude polysaccharides are extracted from *Dendrobium officinale* by removing low-molecular-weight substances such as oligosaccharides, amino acids, lipids, and pigments by extraction with organic solvents [21]. Common extraction methods for crude DOPs include hot water extraction (HWE), ultrasound-assisted extraction, microwave-assisted extraction, enzyme-assisted extraction, steam-assisted flash extraction, mechanochemical-assisted extraction, and enzyme-assisted extraction with deep eutectic solvents [4,20,21,22,23].

HWE is the most commonly used extraction method in laboratories and industry because it is simple, convenient, and safe. However, HWE is associated with tedious extraction and low efficiency. Extraction conditions are the main factors affecting polysaccharide yield in HWE. Wang et al. [18] optimized the extraction process of DOPs with a liquid to material ratio of 24.6:1, an extraction time of 2.1 h, and an extraction temperature of 67.4 °C, resulting in a yield of DOPs of 34.51% and a total polysaccharide content of 90.82% with an extraction efficiency of 92.42%. 

To improve extraction efficiency, techniques such as ultrasound-assisted extraction and microwave-assisted extraction have also been used for the extraction of DOPs. He et al. [4] compared five extraction methods, namely HWE, cold pressing, freeze–thaw cold pressing, ultrasound-assisted hot water extraction, microwave-assisted hot water extraction, and enzyme-assisted hot water extraction, under the same conditions to evaluate their extraction yield and structure. Ultrasonic-assisted hot water extraction and freeze–thaw cold pressing showed higher extraction yields than the other methods, while cold pressing and freeze–thaw cold pressing resulted in polysaccharides with higher molecular weight [4]. Although ultrasonic and microwave techniques can increase extraction efficiency by damaging cell walls and facilitating the release of intracellular substances, prolonged application can cause structural damage to polysaccharides and affect their solubility, thermal stability, and swelling properties [24,25]. In mechanochemical-assisted extraction, small ceramic beads are mechanically vibrated and rotated at high speed to rupture the plant cells and rapidly release the active components into the solvent. This method significantly improves the extraction efficiency and shortens the extraction time. Ma et al. [26] successfully extracted polysaccharides from *Dendrobium officinale* using water as a solvent with a simple, fast (40 s), and high-yield extraction method. Steam-assisted flash extraction is a fast, low-temperature extraction technique that can be carried out at room temperature. The optimal extraction process for DOPs using steam-assisted flash extraction includes a liquid/material ratio of 30 mL/g, a 30-min soaking time, and three extractions at room temperature of 2 min each, resulting in the highest yield of 26.3% [27]. Compared to physically assisted extraction methods, enzyme-assisted extraction has the advantages of mild reaction conditions and high efficiency. Currently, enzymes such as cellulase, pectinase, and protease are mainly used for the processing of polysaccharides. Enzymes can soften the cell walls, change their permeability, and improve the dissolution of cell contents, thereby increasing the efficiency of extraction [23]. In addition, enzyme-assisted extraction based on deep eutectic solvents is a promising method with the advantages of stable performance and fewer environmental problems [22].

The known DOPs are mostly heteropolysaccharides, and research has mainly focused on the composition and molecular weight of monosaccharides. DOPs are mainly composed of glucose (Glup), mannose (Manp), galactose (Galp), xylose, arabinose (Arap), and rhamnose [28]. The molar ratio of mannose to glucose varies depending on the extraction method [4]. According to the available literature, the molecular weight of DOPs ranges from 1.318 to 1930 kDa [29,30,31]. Some extraction methods, such as ultrasound-assisted extraction, can break the main chain of polysaccharides and reduce their molecular weight [4]. Table 1 gives an overview of the extraction methods for DOPs and their structure.

**Figure 1:**
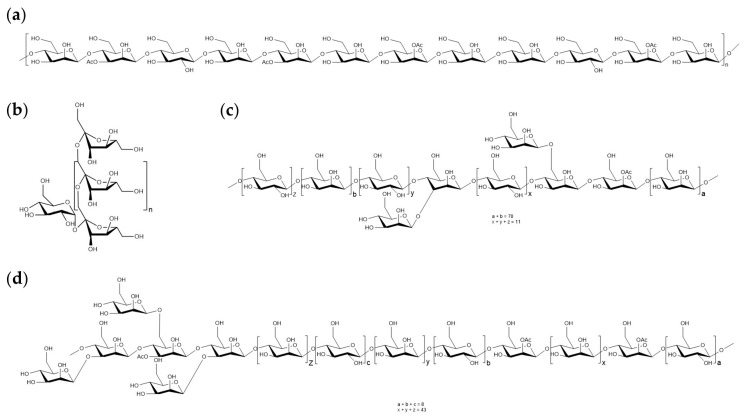
The chemical structure of inulin and DOPs: (**a**) inulin; (**b**) presumed structures of DOP [35]; (**c**) presumed structures of DOPW-1 [36]; (**d**) presumed structures of DOPW-2 [36].

The basic structure of the purified DOPs was elucidated by analyses such as high-performance gel permeation chromatography, Fourier transform infrared spectroscopy, gas chromatography, methylation analysis, and nuclear magnetic resonance. Currently, most DOPs have been identified as glucomannans with 1,4-β-D-Manp and 1,4-β-D-Glcp units, which are acetylated to varying degrees, with or without branching [20]. Due to their high water absorption capacity, glucomannans disperse in water to form highly viscous solutions that are difficult to digest and absorb in the stomach or small intestine [32]; these showed that orally administered DOPs and fluorescently labelled DOPs were neither absorbed nor digested in mice and in vitro models, but were rapidly degraded to SCFAs in the large intestine [7,8]. The classical prebiotic inulin also has similar digestion-resistant properties, thanks to the polysaccharide linked to the end of the glucose envelope by β (2 → 1) bonds of D-fructose [33,34]. The structures that differ between DOPs and inulin are shown in Figure 1.

**Table 1:** The characteristics and extraction of DOPs.

Compound	Extraction Methods	Yield/%	Monosaccharide Compositions	Structures	Mw/Da	Ref.
HDOP	70 °C deionized water extraction, 75% ethanol precipitation	19.12%	Manp/Glup = 6.9:1.0	Backbone chain: →4)-2,6-di-O-acetyl-β-d-Manp-(1→, →4)-3,6-di-O-acetyl-β-d-Manp-(1→, and →4)-6-O-acetyl-β-d-Manp-(1→	3.12 × 10^5^	[37]
DOP	70~75 °C hot-water extraction and 85% ethanol precipitation	Unknown	Manp/Glup/Arap/Galp = 6.2: 2.3:2.1:0.1.	Backbone consisting of 2-O-acetyl-Manp and (1→4)-linked-β-d-Manp and (1→4)-linked-β-d-Glcp residues	8.5 × 10^3^	[38]
DOPs	80 °C distilled water extraction, 75% soluble component	2.76%	Manp/Glup = 5.78:1	Backbone consisting of α-(1→3)-Glcp, branch consisting of α-(1→4)-Glcp and β-(1→4)-Manp	4.56 × 10^3^	[30]
DOPA-1	90 °C deionized water extraction, 85% ethanol precipitation	Unknown	Manp/Glup/Galp = 1:0.42:0.27	Backbone consisting of (1→3), (1→2), and (1→6) linkages.	2.29 × 10^5^	[39]
DOP-1-A1	100 °C distilled water and 1% polyvinylpyrrolidone extraction, 60% alcohol precipitation	3.16%	Manp/Glup/Arap = 40.2:8.4:1.0	Backbone consisting of (1→4)-linked β-d-Manp and β-d-Glcp (in 6:1 ratio)	1.30 × 10^5^	[29]
DOPa	100 °C deionized water extraction, 71.25% ethanol precipitation	Unknown	Manp/Glup = 5.6: 1.0	Backbone consisting of β-(1→4)-D-Manp, β-(1→4)-D-Glcp residues	8.1 × 10^5^	[40]
DOPS-1	Boiling water extraction, 80% ethanol precipitation	Unknown	Manp/Glup/Galp = 3.2: 1.3: 1.0	Backbone consisting of (1→4)-β-D-Glcp, (1→4)-β-D-Manp and 2-O-acetyl-(1→4)-β-D-Manp	1.53 × 10^6^	[41]
DOP-W3-b	100 °C deionized water extraction, 80% ethanol precipitation	1.59%	Manp/Glup = 4.5:1.0	Backbone consisting of β-(1→4)-D-Manp, β-(1→4)-D-Glcp, and β-(1→3,6)-D-Manp residues; branch consisting of β-(1→4)-D-Manp, β-(1→4)-D-Glcp, and terminal β-D-Glcp, and O-acetyl groups attached to O-2 of β-(1→4)-D-Manp	1.543 × 10^4^	[42]
DOP1-DES	Deep eutectic solvents extraction	Unknown	Manp/Glup = 2.2:1.0	Backbone consisting of (1→4)-β-D-Manp and (1→4)-β-D-Glcp	2.98 × 10^5^	[22]
DO	Mechanochemical-assisted extraction	Unknown	Unknown	Unknown	0.66~6.19 × 10^5^	[26]
FP	Flash Extraction	26.3%	Manp/Glup = 3.75~25.60:1	Unknown	Unknown	[27]
DOPCP	Cold pressing	13.79%	Manp/Glup = 3.71:1	Unknown	3.78 × 10^5^	[4]
DOPFTCP	Freeze–thawing cold pressing	20.33%	Manp/Glup = 3.63:1	Unknown	3.69 × 10^5^	[4]
DOPUHWE	Ultrasonic-assisted hot water extraction	20.55%	Manp/Glup = 2.29:1	Unknown	1.97 × 10^5^	[4]
DOPMHWE	Microwave-assisted hot water extraction	17.74%	Manp/Glup = 3.57:1	Unknown	3.24 × 10^5^	[4]
DOPEHWE	Enzyme-assisted hot water extraction	18.50%	Manp/Glup = 2.01:1	Unknown	2.98 × 10^5^	[4]

## 3. *Dendrobium officinale* Polysaccharides–Host Interactions: Health Benefits, Cellular Mechanisms, and the Structure–Function Relationship

### 3.1. Metabolism Regulations

Excessive calorie intake can lead to abnormalities in total cholesterol (TC), triglycerides (TG), low-density lipoprotein cholesterol (LDL-C), and high-density lipoprotein cholesterol (HDL-C) and can contribute to metabolic disorders such as obesity and type 2 diabetes. These metabolic disorders are associated with various diseases, including obesity, type 2 diabetes, hyperlipidemia, hypertension, non-alcoholic fatty liver disease [43], and osteoporosis [44,45]. They can also lead to cancer and cognitive and memory impairment [46,47]. DOPs have been found to lower fasting blood glucose (FBG), insulin resistance, TC, TG, and LDL-C levels while increasing HDL-C levels. The underlying mechanisms of their action could be: (1) bypassing human gastric and small intestinal digestion and utilization by the gut microbiota [5,6,7,8], (2) influencing lipid metabolism and cholesterol homeostasis, reducing cholesterol absorption and utilization [13,48], (3) influencing glucose metabolism, reducing the activity of glucose-metabolizing enzymes, and influencing intestinal function and hormone secretion in the intestine [12,14,49], or (4) influencing protein metabolism, increasing taurine content, and inhibiting the increase in branched-chain amino acids due to insulin resistance [10,12,15,50].

DOPs are mainly completely fermented in the large intestine and form SCFAs such as acetate, propionate, and butyrate. Intervention with DOPs in male C57BL/6 mice exposed to an 11-week high-fat diet (HFD) improved metabolic disturbances, including fatty acid biosynthesis, glutamine metabolism, butyrate metabolism, glutathione metabolism, and the citric acid cycle. Moreover, the effects mediated by DOPs could be partially transferred by transplanting the fecal microbiota into HFD-induced obese mice, suggesting that DOPs exert their metabolic regulatory effects mainly by modulating the composition of the gut microbiota and regulating the production of SCFAs [12].

Mitochondria play a critical role in maintaining cellular metabolic homeostasis, and mitochondrial dysfunction is a common pathologic mechanism underlying diabetes and its complications [51]. In C57BL/6J mice, the potential cellular mechanisms by which DOPs regulate host metabolism are thought to be upregulation of uncoupling protein 1 (UCP1) in mitochondria, activation of peroxisome proliferator-activated receptor-gamma coactivator 1-alpha (PGC-1α) in brown adipose tissue, regulation of thermogenesis and degradation of excess energy, enhancement of insulin signaling pathways and hepatic glycogen synthesis, and improvement of glucose homeostasis [12]. DOPs also improve mitochondrial dysfunction by increasing the activity of mitochondrial respiratory chain complexes, upregulating phosphorylated AMP-activated protein kinase (p-AMPK)/AMPK, and improving tricarboxylic acid cycle function [52]. In addition, DOPs activate the PI3K/Akt pathway to interfere with glycogen synthesis and glucose metabolism [14], mediate PKA and Akt/FoxO1 pathways to further promote hepatic glycogen synthesis, inhibit glycogen degradation, and suppress hepatic gluconeogenesis [49], and regulate the peroxisome proliferator-activated receptor (PPAR) to enhance lipid metabolism [13]. The performance of DOP in metabolism is summarized in Table 2. 

The regulatory effects of DOPs on the host’s metabolism vary depending on the structural factors. A four-week intervention with DOPs and two other natural acetylated glucomannans improved the metabolic syndrome of glucose and lipids in male Wistar rats with streptozotocin-induced type 2 diabetes. In these interventions, there was a positive correlation between molecular weight (Mw) and the hypolipidemic and hypoglycemic effects, while the ratio of mannose to glucose residues showed an opposite trend. The degree of acetylation showed a negative correlation with renal and hepatic function. Both Mw and degree of acetylation were negatively correlated with significant changes in serum carbohydrates. DOPs with a lower molecular weight and degree of acetylation showed greater efficacy in balancing the urea cycle and amino acid metabolism [10,15]. Two types of DOPs exhibited similar glycosidic linkages but differed in their molecular weight and monosaccharide composition. Interestingly, they showed consistent secretion of glucagon-like peptide-1 (GLP-1) and hypoglycemic effects in male streptozotocin-induced diabetic SD mice when administered at the same dose [35].

The performance of DOPs in metabolic disorders is excellent, and it is safe and has no toxic side effects in the treatment of obese mice at a dosage of 1 g/kg/d for 11 weeks [12]. However, DOPs are currently often combined with chemically synthesized drugs (Metformin, etc.). In order to compare the effects, a long intervention period is required, the required dose is high, and the effect is dose-dependent. There is a lack of comparison with natural products such as inulin as a potential prebiotic, and high-quality clinical studies are needed to test them as a potential prebiotic.

**Table 2:** The role and mechanisms of DOPs in the regulation of metabolism.

Substance	Object	Dosage and Duration	Health Outcome	Potential Mechanism	Ref.
Unknown	HFD-induced obese male KM mice	350 mg/kg/d intervention for 8 weeks	Improve intestinal mucosal barrier function.	Regulate gut microbial composition, carbohydrate metabolism, and SCFAs.	[53]
Manp/Glup/Galp = 59.23:35.82:1.61 Mw = unknown	HFD-induced obese male C57BL/6 mice	1.0 g/kg/d intervention for 11 weeks	Alleviate obesity and hepatic steatosis; improve insulin pathway, hepatic glycogen synthesis, and glucose homeostasis; upregulate energy metabolism; increase acetic acid and taurine.	Increase the abundance of beneficial bacteria and SCFAs; upregulate UCP1, PGC-1α, Gck, Gys2, ZO-1, and occludin; downregulate Pck1 and LPS.	[12]
Manp/Glup/Arap = 5.5:1:0.12 Mw = 393.8 kDa	HFD-induced obese male C57BL/6 and ob/ob mice	150 mg/kg/d intervention for 3 months	Improve lipid metabolism, insulin resistance, and purine metabolism; increase glucose tolerance.	Unknown.	[13]
Manp/Glup = 6.9:1 Mw = 312 kDa	STZ-induced type 2 diabetic male Wistar rats	20, 40, 80, 160 mg/kg/d intervention for 8 weeks	Improve lipid metabolism, insulin resistance, and the metabolism of fatty acid, glycerolipid, sphingolipid, phospholipid, bile acid, and amino acid; decrease oxidative stress.	Upregulate GLP-1.	[50]
Manp/Glup =1.38:1.00 Mw = 395 kDa	STZ-induced type 2 diabetic male Balb/c mice	100, 200, 400 mg/kg/d intervention for 4 weeks	Improve hyperglycemia, lipid metabolism, and insulin resistance.	Activate the PI3K/Akt signaling pathways; upregulate IRS1, PI3K, Akt, GS, GLUT4, IR-β, and IR-β; downregulate PPAR-γ, JNK, GSK-3β, and PTP 1B.	[14]
Manp/Glup = 6.9:1 Mw = 312 kDa	STZ-induced type 2 diabetic male Wistar rats	160 mg/kg/d intervention for 4 weeks	Improve hyperglycemia, lipid metabolism, and insulin resistance; normalize the glomerular structure and function in diabetic nephropathy; decrease serum uric acid, urea, and Crea-J.	Improve the metabolic of purine, aldehyde/acetate, purine, tyrosine, bile acid biosynthesis, pyrimidine, glycine/serine, gluconeogenesis, amino sugars, citric acid cycle, and aspartate.	[15]
DOPS-1	HFD-induced type 2 diabetic male Wistar rats	Unknown	Improve hyperglycemia, insulin resistance, and the metabolic disorders of branched-chain amino acids, saccharides, cholic acids, nucleotides, carnitine, indoles, and lipids.	Improve branched-chain amino acid metabolism by decreasing the microbial abundance of valine, leucine, and isoleucine.	[10]
Manp/Glup = 4.41:1.00 Mw = 190 kDa	HFD/STZ-induced type 2 diabetes in male C57BL/6J mice	100, 200, 400 mg/kg/d for 4 weeks	Improve hyperglycemia, insulin resistance, hepatic glycogen synthesis, the stability of hepatic glycogen structure, and liver glucose metabolism; inhibit hepatic glycogenolysis and gluconeogenesis.	Upregulate GS and Akt/Fox01; downregulate G6Pase, PEPCK, cAMP-PKA, and GP.	[49]
Manp/Glup = 6.9:1 Mw = 312 kDa	STZ-induced type 2 diabetes in male Wistar rats	20, 40, 80, 160 mg/kg/d for 8 weeks	Improve the metabolism disorders of methionine, fatty acid, triglyceride, glycerophospholipid, sphingolipid, bile acid, and carbohydrate; normalize levels of dipeptides, hemolytic phospholipids, salicylates, and others.	Unknown.	[48]
Manp/Glup/Arap/Galp = 1.0:1.5:3.5:1.2 Mw = unknown	Alloxan-induced diabetes in male ICR mice	40 mg/kg/d for 3 weeks	Improve hyperglycemia; increase the serum insulin.	Unknown.	[54]
The content of Glup, Manp, Galp, and GalA is 56.24%, 18.68%, 3.67%, 1.29% Mw = 312 kDa Uronic acids = 3.02%	HFD-induced type 2 diabetes mellitus in male C57BL/6J mice	200 mg/kg/d for 8 weeks	Improve hyperglycemia, lipid metabolism, and insulin resistance; improve mitochondrial function in the brain cortex, inhibit apoptosis of brain neurons, and enhance the tricarboxylic acid cycle in the brain cortex; decrease oxidative stress.	Upregulate Bcl-2, Bcl-2/Bax, 5hmC, 5fC, TET2, and p-AMPK/AMPK; downregulate cleaved caspase 3/caspase 3.	[52]
Manp/Glup = 5.18:1 and 4.78:1 Mw = 6.8 kDa and 14.3 kDa	STZ/HFD-induced diabetes in male SD mice	25 and 100 mg/kg/d for 28 days	Improve hyperglycemia; increase the serum insulin.	Upregulate GLP-1; regulate the Ca^2+^/CaM/CaMKII and MAPK signaling pathways.	[35]
Manp/Glup = 1.9:1.0 Mw = unknown	Excessive alcohol consumption and HFD-induced metabolic hypertension in male SD rats	200 mg/kg/d for 7 weeks	Decrease the blood pressure; improve lipid metabolism, intestinal barrier, and endothelial function.	Regulate intestinal microbial composition and SCFAs; activate the SCFA-GPCR43/41 pathways; upregulate occludin, claudin, ZO-1, GPCR41, GPCR41/43, aortic eNOS, and serum NO.	[55]
The content of Manp and Glup is 59.19 and 830.98 mg/g Mw = 8.404 kDa	Difenoxin or deprivation of water-induced constipation in ICR mice	29, 57, 114 mg/kg once a time	Improve constipation; increase intestinal transit rate; facilitate stool evacuation characteristics.	Upregulate motilin, gastrin, acetyl cholinesterase, and substance P; downregulate somatostatin.	[56]
Manp/Glup = 69.70:30.30 Mw = 731 kDa	HFD/STZ-induced type 2 diabetes mellitus in male C57BL/6J mice	120 mg/kg/d for 8 weeks	Improve hyperglycemia, lipid metabolism, insulin resistance, and tissue damage; decrease oxidative stress.	Regulate gut microbial composition.	[57]

### 3.2. Inflammation Modulation

DOPs show significant benefits in the prevention and treatment of chronic inflammation in organs such as the intestines, liver, lungs, and brain. They can increase the level of the anti-inflammatory cytokine IL-10 in serum and in the affected organs and at the same time reduce the level of the pro-inflammatory cytokine TNF-α [17,48,58,59]. In the context of LPS-induced ileitis in male C57BL/6 mice, DOPs show systemic anti-inflammatory effects by suppressing the Th1 cell response in both serum and spleen, comparable to the effects of equimolar administration of inulin [6].

The anti-inflammatory effect of DOPs on the host depends on their structural factors. DOPs could activate the mucosal immune system and prevent intestinal damage caused by dextran sulfate sodium (DSS). The synergistic effects of the glucan-to-mannan ratio, acetyl content, and Mw may influence their functional activities. It has been found that a high Mw-glucan and an extremely high glucan-to-mannan ratio can have negative effects on the beneficial properties of DOPs. Compared to two structurally similar glucans, DOPs have an optimal protective effect [60]. DOPs fragments, which consist of (1→4)-β-D-glucose and (1→4)-β-D-mannose, are formed by enzymatic reactions with highly specific endo-β-1,4-mannanase. These fragments, which resemble intact DOPs prior to the enzymatic reactions, show a significant reduction in the release of pro-inflammatory cytokines. Further studies have identified effective anti-inflammatory fragments of DOPs [61]. The performance of DOPs in regulating inflammation is summarized in Table 3.

DOPs have been extensively studied to determine their anti-inflammatory mechanisms. The current research findings can be summarized as follows:

(1) DOPs exert anti-inflammatory effects by modulating the composition of the gut microbiota and promoting the secretion of SCFAs. Transplantation of the fecal microbiota has shown that the anti-inflammatory, antioxidant and stress-relieving effects of DOPs are transferable to obese mice induced by HFD [12].
(2) DOPs restore intestinal barrier function and protect the intestinal mucosal barrier. In animal models, DOPs enhance the expression of intestinal epithelial tight junction proteins such as occludin and ZO-1, thereby maintaining a stable mucosal barrier, strengthening intestinal barrier function, reducing lipopolysaccharide (LPS) translocation, and lowering serum LPS levels [62,63]. In addition, DOPs reduce the expression of the LPS binding site toll-like receptor 4 (TLR4), inhibit the NF-κB signaling pathway, and reduce the secretion of inflammatory cytokines [58,63].
(3) DOPs alleviate intestinal inflammation by promoting the packaging of miR-433-3p into extracellular vesicles (EVs). Upregulation of miR-433-3p was observed in a DSS-induced colitis mouse model, and DOPs were found to regulate hnRNPA2B1 to facilitate the engulfment of miR-433-3p into gut-derived small EVs (sEVs) in Caco2 cells. Subsequently, these sEVs are transported to LPS-induced macrophages in the lamina propria of the intestine, where miR-433-3p targets the MAPK8 gene, inhibits MAPK signaling pathways, and decreases the secretion of TNF-α and IL-6 by macrophages [64].

In addition to the above conclusions, DOPs have been shown in animal models to activate the Nrf-2/HO-1 signaling pathway in damaged cells at the sites of inflammation, thereby alleviating oxidative damage at the sites of inflammation [58,65,66,67,68].

In summary, it can be said that DOPs perform well in regulating inflammation and have an equivalent inflammation-regulating effect to the classic prebiotic inulin at the same amounts [6]. A dose of 1.2 g three times a day over a period of 12 weeks is safe in the treatment of patients with chronic obstructive pulmonary disease [16]; follow-up studies should improve the quality of population experiments and investigate the efficacy of DOPs in regulating inflammation.

**Table 3:** Role and mechanism of DOP in inflammatory regulation.

Substance	Object	Dosage and Duration	Health Outcome	Potential Mechanism	Ref.
Manp/Glup/Arap = 5.5:1:0.12 Mw = 393.8 kDa	HFD-induced obesity in male C57BL/6 mice and ob/ob mice	150 mg/kg/d for 3 months	Improve insulin resistance and visceral adipose tissue inflammation.	Unknown.	[13]
Manp/Glup = 6.9:1 Mw = 312 kDa	STZ-induced type 2 diabetes in male Wistar rats	20, 40, 80, 160 mg/kg/d for 8 weeks	Improve proliferation of adipocytes; decrease inflammatory infiltration, glycoprotein deposition on capillary basement membrane, and oxidative stress in adipose tissue cells.	Unknown.	[50]
Manp/Glup/Arap Mw = 393.8 kDa	Osteoporosis in elderly mice	150 mg/kg/d for 3 months	Increase bone formation rate and mineral absorption; decrease bone marrow adipose tissue accumulation and oxidative stress.	Activate the Nrf-2 signaling pathways; upregulate Nrf-2 and HO-1.	[65]
Manp/Glup = 6.9:1 Mw = 312 kDa	STZ-induced type 2 diabetes in male Wistar rats	20, 40, 80, 160 mg/kg/d for 8 weeks.	Improve hepatic oxidative stress and inflammation.	Unknown.	[48]
Manp/Glup = 6.9:1 Mw = 312 kDa	LPS-induced enteric inflammation in male C57BL/6 mice	160 mg/kg/d for 2 weeks	Inhibit Th1 cell responses in serum and spleen, exerting systemic anti-inflammatory effects; induce Th17 cell differentiation in spleen and mesenteric lymph nodes.	Upregulate Rorc; down-regulate Foxp3, Tbx21, and HIF-1.	[6]
Unknown	STZ-induced diabetic cataracts in male Wistar rats	0.1 g/kg two times a day for 12 weeks	Decrease the severity of diabetic cataracts.	Inhibit The MAPK signaling pathways; downregulate ERK1, Raf, Ras, and MiRNA-125b.	[69]
Manp/Glup = 1.9:1.0 Mw = unknown	Excessive alcohol consumption and HFD-induced metabolic hypertension in male SD rats	200 mg/kg/d for 7 weeks	Improve lipid metabolism, intestinal barrier, and endothelial function; alleviate hepatic inflammation lesions and ameliorate fatty inflammation.	Regulate gut microbial composition and SCFAs; activate the SCFA-GPCR43/41 signaling pathways; upregulate GPCR41, GPCR41/43, occludin, claudin, ZO-1, aortic eNOS, and serum NO.	[55]
The content of Manp and Glup is 57.3 mg/g and 670.2 mg/g	40 cases of moderate chronic obstructive pulmonary disease patients in China	1.2 g three times a day for 12 weeks	Improve serum and pulmonary inflammation; increase patient lung capacity to improve pulmonary function.	Upregulate aquaporin-5; downregulate mucin-5AC.	[16]
The content of Manp and Glup = 57.3 mg/g and 670.2 mg/g	Passive smoking models in male SD rats	50, 200 mg/kg/d for 28 days	Alleviate infiltration of inflammatory cells in lung tissue; decrease lymphocyte and monocyte counts in serum and oxidative stress in lung.	Inhibit the ERK, p38 MAPK, and NF-κB signaling pathways; downregulate MCP-1 and CINC-1.	[17]
Manp and Glup in a molar percent of 71.2% and 98.1%	Bleomycin-induced pulmonary fibrosis in male SD rats	200 mg/kg/d for 28 days	Improve pulmonary fibrosis and inflammation; reduce collagen deposition; decrease the transformation of rat alveolar epithelial type 2 cells into myofibroblasts.	Inhibit the TGFβ1-Smad2/3 signaling pathways; downregulate serum TGFβ1, Smad2/3, pSmad2/3, collagen I, and fibronectin protein expression.	[70]
Manp and Glup = 3.8:1.0 Mw = 132 kDa	Ethanol-induced gastric mucosal injury in male Sprague Dawley rats	124, 248 mg/kg/d for 7 days	Improve gastric mucosal injury; inhibit ethanol-induced mucosal protein loss and cell apoptosis.	Upregulate Bcl-2; downregulate Bax.	[71]
Manp and Glup = 3.8:1.0 Mw = 132 kDa	H_2_O_2_-induced HFE145 cells	50 μg/mL and 500 μg/ml	Protect cells and inhibit apoptosis.	Activate PPAR signaling pathways; downregulate p-NF-κBp65/NF-κBp65, Bax, and cleaved caspase 3; upregulate Bcl-2.	[71]
Manp/Glup/Arap = 5.55:1:0.12 Mw = 393 kDa	DSS-induced colitis in male Balb/c mice	50, 100, 200 mg/kg/d for 7 days	Improve colitis-induced lung injury and pulmonary edema; decrease inflammatory cell infiltration, inflammatory response, and oxidative stress.	Activate the Nrf-2 signaling pathways; upregulate HO-1, NQO-1, and ZO-1; downregulate Ly6G and TLR4.	[58]
Manp/Glup/Arap = 5.55:1:0.12 Mw = 393 kDa	DSS-induced ulcerative colitis in male Balb/c mice	50, 100, 200 mg/kg/d for 7 days	Improve inflammation and histopathological changes; inhibit neutrophil infiltration, splenomegaly, and thymic atrophy to restore the immune system damage.	Inhibit the NLRP3 inflammasome signaling pathways; downregulate Ly6G, NLRP3, ASC, and caspase 1.	[72]
Manp/Glup/Arap = 5.55:1:0.12 Mw = 393 kDa	DSS-induced acute colitis and secondary hepatic injury in male Balb/c mice	50, 100, 200 mg/kg/d for 14 days	Improve dyslipidemia; inhibit infiltration of inflammatory cells into hepatic macrophages; decrease oxidative stress and liver damage.	Activate the Nrf-2 signaling pathways; upregulate Nrf-2, HO-1, and NQO-1.	[67]
Manp/Glup = 4.76:1.00 Mw = 2.921 kDa And Manp/Glup = 4.44:1.00 Mw = 141.2 kDa	DSS-induced colitis in male Balb/c mice	200 mg/kg/d for 7 days	Improve the clinical symptoms of colitis, mucosal damage, and inflammatory cell infiltration; inhibit splenomegaly caused by colitis and inflammation.	Activate the GPR41/43 signaling pathways; upregulate GPR41, GPR43, the abundance of gut microbiota, acetic acid, i-butyric acid, and total SCFAs.	[61]
Manp/Glup = 4.17:1 Mw = 618.029 kDa	DSS-induced colitis in female Balb/c mice	200 mg/kg/d for 20 days	Improve intestinal microenvironment homeostasis; inhibit inflammatory cell infiltration in the intestinal lamina propria and intestinal inflammation.	Upregulate MiR-433-3p.	[64]
Manp/Glup = 6.9:1 Mw = 312 kDa	DSS-induced colitis in female BALB/c mice	200 mg/kg/d for 18 days	Improve the clinical symptoms of colitis; increase thymus index, colon length, crypt depth, intestinal wall thickness, and intestinal mucosal integrity; repair colonic mucosal damage; decrease inflammatory reactions.	Downregulate TLR-2, TLR-4, TLR-6, and TLR-9.	[60]
Manp/Glup = 6.9:1 Mw = 312 kDa	Healthy female Balb/c mice	40, 80, 160 mg/kg /d for 10, 20, 30 days	Maintain colon health; increase in colon length and in fecal water content; decrease in defecation time.	Improve the fermentation and regulation of the colon microenvironment; upregulate acetic acid, n-butyric acid, propionic acid, isovaleric acid, i-butyric acid, n-valeric acid, and total SCFAs.	[73]
Manp/Glup/Gal = 59.23:35.82:1.61 Mw = unknown	HFD-induced obesity in male C57BL/6 mice	1.0 g/kg/d for 11 weeks	Improve intestinal barrier function and systemic anti-inflammatory activity; decrease inflammation and oxidative stress.	Upregulate the abundance of beneficial bacteria, ZO-1, and occludin; downregulate NOX2 and NOX4.	[12]
Unknown	Anhydrous ethanol-induced gastric ulcer in male SD rats	0.12, 0.23, 0.46 g/kg/d for 1 week	Improve gastric epithelial defects caused by gastric mucosal injury; inhibit inflammatory cell infiltration and inflammation.	Inhibit the MAPK signaling pathways; downregulate MEK1, MEK, ERK1, and Raf-2; upregulate EGFR and TFF1.	[74]
Manp/Glup/Arap = 5.55:1:0.12 Mw = 393 kDa	Ovariectomy- and galactose-induced learning and memory deficits in female Kunming mice	140 mg/kg/d for 3 months	Alleviate neuroinflammation and oxidative stress. Improve learning and memory deficits and hippocampal neuronal cells; inhibit activation of astrocytes and microglia.	Activate the Nrf-2/HO-1 signaling pathways; upregulate Nrf-2 and HO-1; downregulate GFAP and Iba-1.	[68]
Unknown	Senescence-accelerated mouse susceptible male 8 mice and control senescence-accelerated male mouse resistant 1 cognitive impairment	40 mg/kg/d for 3 months	Improve cognitive and inflammation; modulate microglia activation; increase transition from M1 to M2 phenotype and Aβ degradation to ameliorate aberrant phosphorylation of Tau and Aβ accumulation.	Upregulate NEP, IDE, BDNF, NGF, and PSD95.	[59]
Manp/Glup/Arap = 5.55:1:0.12 Mw = 393.8 kDa	Female Kunming naturally aging mice	70 mg/kg/d for 10 weeks	Improve mitochondrial activity; decrease inflammatory reactions, oxidative stress, and pathological damage to the ovary; increase the number of follicular cells in different stages of the ovary.	Upregulate Bcl-2 and estradiol; downregulate IL-12p70, p53, and p-p65.	[75]
Manp/Glup = 2.55:1.00 Mw = 746.52 kDa	Cognitive impairment in male C57BL/6J mice due to circadian rhythm disruption	200 mg/kg/d for 4 weeks	Improve gut microbial disorders and mucosal integrity; inhibit hippocampal neuronal damage and inflammatory cell infiltration.	Regulate gut microbial composition; upregulate ZO-1, occludin, eletriptan, moclobemide, and paliperidone; downregulate Aβ.	[62]
Mw = 8.551 kDa	Acetaminophen-induced liver injury in male ICR mice	50, 100, 200 mg/kg/d for 30 days	Improve liver damage; decrease inflammatory infiltration and oxidative stress.	Activate the Nrf-2 signaling pathways; upregulate GCLC, GCLM, HO-1, and NQO1; downregulate Keap1.	[66]
Manp/Glup = 4.41:1.00 Mw = 195 kDa	CCl4-induced liver fibrosis in male SD rats	200, 400, 800 mg/kg/d for 8 weeks	Maintain intestinal homeostasis; improve intestinal mucosal barrier; decrease intestinal cell permeability and apoptosis; decrease inflammation and prevent hepatic fibrosis.	Upregulate ZO-1, Bcl-2, occludin, and claudin-1; inhibit the LPS-TLR4-NF-κB pathways; downregulate LPS, TLR4, NF-κB, and p-IκBα; downregulate Bax, caspase 3, TGF-β, α-SMA, and collagen I.	[63]
Unknown	PTZ-induced epilepsy in male SD rats	1.5 g/kg/d for 4 weeks	Decrease brain inflammation and seizures; protect brain neurons.	Downregulate the MAPK pathways, p-ERK1/2, p-JNK, and p-p38; upregulate p-MKP-1.	[76]
Unknown	Ovariectomy-induced menopausal depression in female Kunming mice	150, 300, 600 mg/kg/d for 4 weeks	Improve the clinical symptoms of depression; decrease inflammation.	Inhibition of microglia activation; upregulate estradiol; downregulate CRH, ACTH, and corticosterone.	[77]

### 3.3. Immunity Moderation

Animal studies have shown that DOPs can restore the phagocytosis capacity of blood leukocytes and macrophages to normal levels [18,78]. By modulating the balance between Th1 and Th2 responses, DOPs also increase the secretion of cytokines in mice [78,79] and enhance both cellular and humoral immunity [42], thereby exhibiting a significant preventive effect against immunodeficiency induced by certain factors. In healthy animals, DOPs increase the level of nonspecific immunoglobulins, regulate the Th1/Th2 balance, and enhance humoral immune function [80]. Further details on the immunomodulatory effects of DOPs are summarized in Table 4.

**Table 4:** Role and mechanism of DOP in immunity moderation.

Substance	Object	Dosage and Duration	Health Outcome	Potential Mechanism	Ref.
Manp/Glup = 5.59:1 Mw = unknown	Cyclophosphamides-induced immunosuppression in female Kunming mice	40, 80, 160 mg/kg/d for 30 days	Improve cellular immunity, humoral immunity, and phagocytosis of monocytes; increase the quality of immune organs; decrease oxidative stress.	Upregulate CD3, CD4, CD8, CD4/CD8, IL-4, IL-6, IL-10, TNF-α, IgM, IgG, and hemolysin.	[18]
Manp/Glup = 5.16:1.3 Mw = 262.4 ku	Cyclophosphamides-induced immunosuppression in male BALB/c mice	160 mg/kg/d for 7 days	Improve intestinal health, immunomodulation, and the balance between Th1 and Th2 types; increase mass of immune organs.	Upregulate acetic acid, propionic acid, butyric acid, valeric acid, total SCFAs, IFN-γ, TNF-α, and IL-6; downregulate IL-4 and IgE.	[81]
Manp/Glup = 6.9:1 Mw = 312 kDa	Cyclophosphamides-induced immunosuppression in female Balb/c mice	40, 80, 160 mg/kg/d for 7 days	Improve immunomodulation; stimulate plasma cell differentiation, the secretion of immunoglobulin, and cytokines; increase the expression of Th1-type T cells.	Upregulate CD4, CD8 T lymphocytes, T-bet/GATA-3, Pax5, XBP-1, Blimp-1, TNF-α, IFN-γ, IL-4, IgA, IgM, and IgG.	[78]
Manp/Glup = 4.5:1 Mw = 15.43 kDa	Healthy female ICR mice	0.5, 2 g/kg/d for 7 days.	Improve intestinal mucosal structure, intestinal mucosal immunoreactivity, and the balance between Th1 and Th2 types.	Upregulate IFN-γ, IL-4, and IgA.	[42]
Manp/Glup = 6.9:1 Mw = 312 kDa	Cyclophosphamides-induced immunosuppression in male Balb/c mice	40, 80, 160 mg/kg/d for 4 weeks	Improve immunomodulation and proliferation of splenocytes; accelerate phagocytosis by peritoneal macrophages.	Upregulate CD3, CD4/CD8, TNF-α, IL-6, IgM, IgG, and serum hemolysin.	[82]
Glup/GluA = 19.4:1.2 Mw = 39.4 kDa	Healthy female C57BL/6 mice	0.25% DOP aqueous solution instead of drinking water, free water intervention for 25 days	Improve the production of more butyrate by gut microorganisms to participate in immune mediation.	Regulate gut microbial composition; upregulate butyrate, acetic acids, Muc-2, IL-10, TNF-α, IL-1β, and IgM; downregulate fecal lipocalin-2.	[80]

The regulatory effects of DOPs on host immunity vary depending on their structural factors. Wang et al. [18] found that in animal models, the antioxidant activity of the organ is independent of the dosage of DOPs. On the other hand, differently structured DOPs can have dose-dependent effects on the antioxidant capacity of the organism [66]. DOPs modulate the ratio of T lymphocyte subsets and exert immunosuppressive effects, with the advantage of reversing the reduction of CD4 and CD8 T lymphocytes more effectively than the other two different sources of acetylated glucans [78]. DOPs show higher immunomodulatory capabilities compared to the total sum of DOPs from the same source, suggesting that O-acetylated glucan is one of the major bioactive components of DOPs [82]. Shan et al. [83] performed sonication or deacetylation treatment of DOPs, resulting in subgroups with different degrees of acetylation or molecular weights. The polysaccharide components with similar degrees of acetylation but lower molecular weights exhibited stronger immunomodulatory activity in vivo, while low and high degrees of acetylation of DOPs did not result in enhanced immunomodulatory activity, suggesting that a certain range of degrees of acetylation may be a key factor for the immunomodulatory activity of DOPs.

The current state of research on the immunomodulatory effects of DOPs is as follows: (1) DOPs regulate the composition of the gut microbiota and modulate the levels of vitamins, amino acids and SCFAs by promoting the differentiation of regulatory T cells in the intestine to regulate immune responses [80,81,84]; (2) By upregulating the mRNA expression of T-bet and GATA-3, DOPs increase the proportion of CD4 T lymphocytes, increase the secretion of Th1 cytokines, promote the formation of T lymphocyte subsets, and increase the production of non-specific immunoglobulins (IgA, IgM, IgG) [78]. In experiments with the human leukemia monocytic cell line, DOPs induce immune responses via the TLR4-mediated NF-κB signaling pathway. Treatment with TLR4 antagonists and MyD88 inhibitors inhibits the phosphorylation of NF-κB induced by DOPs, suggesting that DOPs can induce immune responses via the TLR4 signaling pathway, which mediates NF-κB activation and generates the chemotactic cytokines CCL4 and IP10 [85]. 

The immune performance of DOPs can enhance immunity, but there are few reports on the research of the mechanism, and further research of the mechanism needs to be conducted into their function as a prebiotic.

### 3.4. Cancer Intervention

The development of cancer is a complex process that is closely linked to immunity, inflammation, and metabolism. The performance of DOPs in cancer is shown in Table 5. Current research on the preventive effects of DOPs in cancer is primarily based on their comprehensive inhibition of cancer progression through modulation of metabolism, inflammation, and immune regulation. (1) In terms of metabolic regulation, DOPs improve mitochondrial function in lymphocytes, increase ATP and glucose levels in tumor-infiltrating lymphocytes [86], and supply activated lymphocytes with ATP and glucose in their anti-tumor response [87], while reducing the expression of PD-1 and enhancing the anti-tumor immune response [86]; DOPs also increase the level of endogenous metabolites, such as betaine, in serum, leading to anti-tumor effects [88]. (2) In terms of inflammatory regulation, DOPs reduce the inflammatory response in colon cancer mice (decreased TNF-α and IL-1β levels), restore the barrier function of the intestinal epithelium (increased ZO-1 and occludin expression), improve the tumor microenvironment, and inhibit tumor growth [86]. (3) In terms of immune regulation, DOPs increase the activity of natural killer (NK) cells and cytotoxic T lymphocytes (CTLs), increase the concentration of non-specific and tumor-specific antibodies (IgG, IgG1, IgG2a, and IgG2b) and suppress tumor growth in transplantation models [89]. (4) DOPs inhibit the Wnt/β-catenin signaling pathway and suppress the proliferation of cancer cells [88], increase the pro-apoptotic factor Bax, decrease the anti-apoptotic factor Bcl-2, and promote apoptosis of tumor cells [90]. In addition, DOPs can activate the Nrf-2/HO-1 signaling pathway and reduce oxidative stress [19].

The anticancer performance of DOPs remains to be developed and the mechanisms remain to be explored. DOPs have the potential to interfere with the cancer process. Many natural polysaccharides have been used in tumor or adjuvant chemotherapy, and the anticancer effect can be enhanced by chemical modification techniques [91]. The use of anticancer polysaccharides in combination with chemotherapeutics and adjuvants for cancer vaccines will be explored in the future.

**Table 5:** Role and mechanisms of DOPs in cancer intervention.

Substance	Object	Dosage and Duration	Health Outcome	Potential Mechanism	Ref.
Manp/Glup/Arap = 5.5:1:0.12 Mw = 393.8 kDa	AOM/DSS-induced colon cancer in male Balb/c mice	50, 100, 200 mg/kg/d for 6 weeks	Improve intestinal epithelial barrier function, intestinal anti-tumor immune response, and inflammation; inhibit the formation and growth of colon tumors.	Upregulate ZO-1, occludin, CD8 CTLs, ATP, and glucose of tumor-infiltrating lymphocytes; downregulate TNF-α, IL-1β, and PD-1.	[86]
Mw = 3.5 and 1000 kDa	MNNG-induced gastric precancerous lesions in male Wistar rats	2.4, 4.8, 9.6 g/kg/d for 7 months	Prevent gastric precancerous lesions; protect gastric mucosa and subsequent liver and kidney damage; improve the weight loss and reduce intestinal epithelial chemotaxis, simultaneously.	Activate the Nrf-2 pathways; upregulate Nrf-2, HO-1, and NQO-1; downregulate 8-OHdG.	[19]
Mw = 3.5 and 1000 kDa	MNNG-induced gastric precancerous lesions in male Wistar rats	2.4, 4.8, 9.6 g/kg/d for 7 months	Modulate endogenous metabolites; ameliorate oxidative stress; inhibit induced gastric precancerous lesions.	Inhibit the Wnt/β-catenin pathways; downregulate Wnt2β, Gsk3β, PCNA, CyclinD1, and β-catenin; upregulate serum endogenous metabolites such as betaine.	[88]

## 4. Relationship between *Dendrobium officinale* Polysaccharides and Gut Microbiota

In vitro and in vivo experiments have shown that the supplementation of DOPs significantly alters the diversity and composition of the gut microbiota and increases the production of primary metabolites such as acetate, propionate, and butyrate [10]. This supplementation has been shown to restore the disrupted gut microbiota and improve microbial diversity in recombinant obese animals [12] or improve the composition of the gut microbiota in model animals, making it more similar to a healthy state. However, these improvements depend on the dose of DOPs and the sex of the host. Thus, low-dose DOPs lead to a slight decrease in the abundance of Bacteroidetes compared to the control group, while high-dose DOPs increase their abundance. In men, there is a slight increase in the relative abundance of Firmicutes and a decrease in Bacteroidetes, while in women, the relative abundance of Bacteroidetes decreases [11]. The increase in the relative abundance of *Lactobacillus* is lower in men than in women, and females show a higher increase in propionate production in their gut microbiota. Therefore, DOPs may have greater health benefits in men than in women [31]. In addition, the changes in the gut microbiota are also influenced by the structural factors of DOPs. For example, DADOP-1 and DADOP-2 are two subgroups of the same DOPs with different degrees of acetylation. DADOP-1 primarily increases the abundance of *Alistipes* and decreases *Helicobacter* in cyclophosphamide-treated mice, while DADOP-2 significantly increases the abundance of *Lactobacillus* and *Akkermansia* [83]. The fragment EDOP, which is produced by enzymatic reactions of DOPs, shows a remarkably similar effect on the abundance of the gut microbiota compared to DOPs. This is further evidence for the assumption that EDOP is the main active component of DOPs [61]. Specific changes in the gut microbiota induced by DOPs both in vitro and in vivo are shown in Table 6.

**Table 6:** DOPs induce changes in gut microbial levels at the genus level.

Structure	Object	Dosage and Duration	The Genus Level of Gut Microbiota		Ref.
			Increase	Decrease	
The content of Manp, Glup, and Galp is 49.03%, 16.54%, 14.83%	In vitro fermentation of healthy human feces	200 mg/15 mL culture fluid for 48 h	*Bacteroides*, *Prevotella*, and *Faecalibacterium*	*Citrobacter*	[5]
The content of Manp and Glup is 61.10% and 36.92% Mw = 277 KDa and 1318 Da	In vitro fermentation of healthy human feces	10 mg/5 mL culture fluid for 24 h	*Bifidobacterium*, *Bacteroides*, *Lactobacillus*, *Enterococcus*, *Streptococcus* (in women), and *Prevotella*_9 (in men)	Lachnospiraceae UCG-004, *Lachnoclostridium Escherichia-Shigella*, and *Paraclostridium*	[31]
Manp/Glup/Galp/Rhap = 59.31:33.31:1.00:0.51 Mw = 291 kDa	In vitro fermentation of healthy human feces	20, 40, 80 mg/10 mL culture fluid for 24 h	*Bifidobacterium*, *Prevotella*_9, *Lactobacillus*, *Faeca-libacterium*, *Pseudobutyrivibrio*, *Pediococcus*, and *Lachnoclostridium*	*Bacteroides*, *Escherichia-Shigella*, *Enterobacter*, *Dialister*, and *ParaBacteroides*	[11]
Manp/Glup/Galp/Rhap = 59.31:33.31:1.00:0.51 Mw = 291 kDa	Healthy male ICR mice	100, 200 mg/kg/d for 21 days	*Lactobacillus*, Desulfovibrionaceae_unclassified, *Klebsiella*, and *Lactococcus*	Ruminococcaceae_unclassified, *Desulfomicrobium*, *Papillibacter*, *Desulfovibrio*, *Pseudomonas*, *Sandaracinobacter*, and *Corynebacterium*	[11]
Manp/Glup = 4.0:1.0 Mw = 277.3 kDa	Healthy male mice	200 mg/kg/for 4 weeks	*Lactobacillus*, *Bifidobacterium*, and *g-Roseburia*	*Proteobacteria*	[92]
Manp/Glup =1.9:1.0 Mw = unknown	Excessive alcohol consumption and HFD-induced metabolic hypertension in male SD rats.	200 mg/kg/d for 7 weeks	Lachnospiraceae_NK4A136_group, *Lactobacillus*, and NK4A214_group	*Blautia*	[55]
Unknown	HFD-induced obese male KM mice	350 mg/kg/d for 8 weeks	Lachnospiraceae_NK4A136_group, *Lactobacillus*, and *Candidatus_Arthromitus*	*Corynebacterium_1* and *Staphylococcus*	[53]
Manp/Glup = 4.76:1.00 Mw = 2.921 kDa And Manp/Glup = 4.44:1.00 Mw = 141.2 kDa	DSS-induced colitis in male Balb/c mice	200 mg/kg/d for 7 days	Ruminococcaceae_UCG—014, *Bacteroides*, and *Lactobacillus*	*Akkermansia*	[61]
Glup/GluA = 19.4:1.2 Mw = 39.4 kDa	Heathy female C57BL/6 mice	0.25% DOP aqueous solution instead of drinking water, free water intervention for 25 days	*Akkermansia muciniphila*, *Ruminococcus*, *Eubacterium*, *Clostridium*, *Bifidobacterium*, and *Parabacteroides*	*Proteobacteria* and *Prevotella*	[80]
Manp/Glup = 2.55:1.00 Mw = 746.52 kDa	Cognitive impairment in male C57BL/6J mice due to circadian rhythm disruption	200 mg/kg/d for 4 weeks	*Akkermansia*, *Alistipes*, and *Dubosiella*	*Desulfovibrio*, *Candidatus*_*Saccharimon*, and *Clostridia*	[62]
Manp/Glup/Galp = 59.23:35.82:1.61 Mw = unknown	HFD-induced obesity in male C57BL/6 mice	1.0 g/kg/d for 11 weeks	*Muribaculum*, *Akkermansia*, and *Bifidobacterium*	*Blautia*, *Lachnoclostridium*, *Bilophila*, and *Mucispirillum*	[12]
Manp/Glup = 6.9:1 Mw = 312 kDa	STZ-induced type 2 diabetes in male Wistar rats	160 mg/kg/d for 4 weeks		*Clostridium*, *Bacteroides*, *Prevotella*, *Klebsiella*, *Escherichia*, *Streptococcus*, and *Staphylococcus aureus*	[10]
Manp/Glup = 69.70:30.30 Mw = 731 kDa	HFD/STZ-induced T2DM in male C57BL/6J mice	120 mg/kg/d for 8 weeks	*Dubosiella*, *Lysinibacillus*, *Ileibacterium valens*, *Faecalibaculum rodentium*, and *Akkermansia muciniphila*	*Lactobacillus reuteri*, *Lactobacillus johnsonii*, *Enterococcus Casseliflavus*, and *Eubacterium Plexicaudatum*	[57]
Manp/Glup = 7.32:1 Mw = 279 kDa	Cyclophosphamides-induced immunosuppression in male BALB/c mice	100 mg/kg/d for 9 days	*Lactobacillus*, *AlloPrevotella*, *Akkermansia*, Ruminococcaceae_UCG-014, and *Alistipes*	*Helicobacter*	[83]

The predominant phyla in the gut microbiota are Firmicutes and Bacteroidetes, which together make up about 90% of the microbial community in the human gut [93]. DOPs primarily cause changes in the abundance of Firmicutes and Bacteroidetes at the phylum level and consistently show a decrease in the Firmicutes/Bacteroidetes ratio [5,12,31,55,62,92]. The ratio of Firmicutes to Bacteroidetes is associated with obesity [94]. It is evident in Table 6 that DOPs mainly affect the abundance of *Lactobacillus*, *Bifidobacterium*, *Akkermansia*, *Bacteroides*, and *Prevotella* at the genus level. Among these, the abundance or ratio of *Prevotella* and *Bacteroides* enterotype and the abundance of *Bifidobacteria* are common features associated with the response to dietary fiber intake [95]. The different digestive modes of the gut microbiota influence their competitive advantage or preference for nutrient acquisition and occupation of the ecological niche in the human gastrointestinal tract, resulting in different microbial community characteristics. Six weeks after infusion of microbiota from lean male donors, the insulin sensitivity of male rehabilitants with metabolic syndrome increased along with the level of butyrate-producing gut microbiota [96]. Oral transplantation of fecal microbiota in combination with supplementation with low-fermentable fiber improved insulin sensitivity in patients with severe obesity and metabolic syndrome [97].

**Figure 2:**
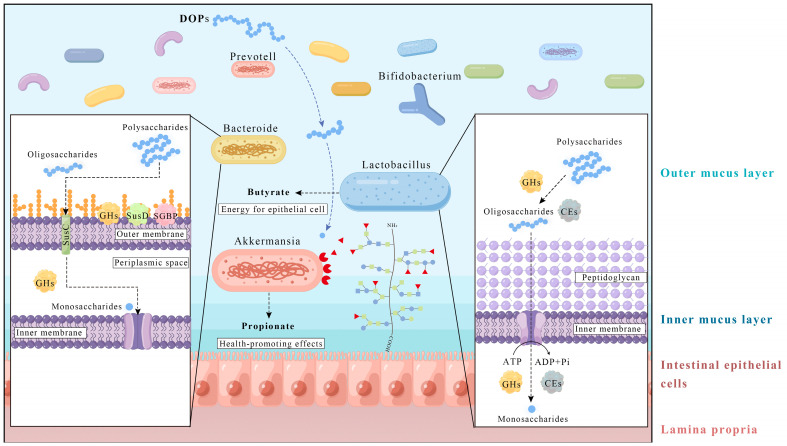
Presumptive relationship between DOP and gut microbes.

(1) *Bacteroides* and *Prevotella*, which belong to the Bacteroidetes, exhibit a remarkable ability to efficiently degrade and metabolize glucomannan and have a positive impact on the growth of microbial communities such as *Lactobacillus* and *Bifidobacterium*. One of the recognized features of *Bacteroides* and *Prevotella* within the Bacteroidetes is their robust polysaccharide degradation system, which includes polysaccharide utilization loci (PULs) that target complex polysaccharides, such as glucomannan, and especially undigested dietary fibers to enhance ecological adaptability [98]. PULs enable the effective utilization of various polysaccharides, especially undigested dietary fibers, to improve ecological adaptability [99]. PULs of *Bacteroides* include starch utilization systems (Sus) and related proteins such as the Ton-B dependent porin (SusC) and the glycan-binding protein (SusD) and their homologs. These proteins, including the glycan-binding surface proteins, are involved in the recognition and binding of target polysaccharides. *Bacteroides* PULs also encode numerous genes for carbohydrate-active enzymes (CAZymes), including glycoside hydrolases (GHs), carbohydrate esterases (CEs), glycosyltransferases, and polysaccharide lyases, which facilitate the depolymerization of target polysaccharides [98,100]. These enzymes play a crucial role in the uptake and utilization of DOPs and other dietary components, as shown in Figure 2. *Bacteroides* ovatus possesses several unique PULs that are capable of degrading semi-fibrous polysaccharides [101]. Within the PULs of *Bacteroides*, the presence of BdPUL12 was observed, which promotes the degradation of β-mannan-like polysaccharides, such as glucomannan, to mannooligosaccharides and mannose. This facilitates the co-cultivation of *Lactobacillus helveticus* and *Bifidobacterium adolescentis*, leading to increased acetate and total SCFA levels [102]. Oral ingestion of *Bacteroides* xylanisolvens DSM 23964 was indeed able to increase the level of natural anti-TFα IgM antibodies in healthy adults and reduce the risk of cancer development [103]. *Bacteroides fragilis* HCK-B3 and *Bacteroides ovatus* support intestinal balance by maintaining the diversity of the gut microbiota and alleviating LPS-induced inflammation by either modulating cytokine production or restoring the Treg/Th-17 balance [104]. In addition, *Bacteroides* has the ability to remove two and/or three O-acetyl groups. *Bacteroides ovatus* harbors precursor carbohydrate esterases that are involved in substrate deacetylation [105]. It has been observed that *Bacillus* and *Bacteroides* found in the human gut can eliminate O-acetyl groups through acetyl-xylan esterases by fermenting O-acetyl-galactomannan in vitro [106]. On the other hand, within the genomic composition of *Prevotella* PULs and CAZymes, three SusD protein clusters are known to target hemicelluloses or pectin [107], leading to the formation of metabolites such as acetate and propionate from the degradation of glucomannan, although variations in the metabolites produced have been observed between different species [108]. *Prevotella histicola*-treated mice with experimental autoimmune encephalomyelitis exhibited an increased frequency and number of CD4+FoxP3+ regulatory T cells in the periphery and gut and a decreased frequency of pro-inflammatory IFN-γ and IL17-producing CD4 T cells in the central nervous system, suggesting that *Prevotella histicola* suppresses the disease by enhancing anti-inflammatory immune responses and inhibiting pro-inflammatory immune responses [109].

In summary, *Bacteroides* and *Prevotella* are able to degrade DOPs and two and/or three O-acetyl groups in the structure of DOPs, produce SCFAs, and are beneficial for the growth of *Bifidobacterium* and *Lactobacillus* microbial communities. 

(2) *Bifidobacterium* and *Lactobacillus* as common probiotic strains are only able to break down and utilize glucomannan to a limited extent. A meta-analysis examined the effects of probiotic interventions (mainly *Lactobacillus* and *Bifidobacterium*) on patients with type 2 diabetes. The results showed that probiotic treatment can reduce glycosylated hemoglobin, fasting blood glucose, and insulin resistance and improve glucose metabolism disorders in patients with type 2 diabetes [110,111,112,113]. A recent study evaluated 25 studies on the potential role of probiotics in the treatment of ulcerative colitis in adults, 21 of which showed that probiotics were effective in achieving or maintaining remission of ulcerative colitis. Supplementation with *Bifidobacterium* spp. or a combination of probiotics containing mainly *Bifidobacterium* spp. and *Lactobacillus* was the most effective intervention [114]. *Bifidobacterium* typically adapts to lower levels of polysaccharide polymerization and uses a similar mode of polysaccharide utilization as the Firmicutes. Apart from the PULs found in Bacteroidetes, Firmicutes encode a number of transport proteins, including ATP-binding cassette transporters, transporters of the major facilitator superfamily, and transporters of the phosphonolpyruvate phosphotransferase system [115]. The uptake and utilization of DOPs and other dietary components by Firmicutes is shown in Figure 2. Fermentation experiments with large amounts of glucomannan as an additional carbon source showed that different *Bifidobacterium* species can produce lactate, acetate, propionate, and butyrate [116]. Glucomannan, which is derived from the enzymatic hydrolysis of cellulose, significantly promotes the growth of *Bifidobacterium* and *Lactobacillus*, while pathogenic bacteria such as *Escherichia coli* cannot grow on a medium in which hydrolyzed glucomannan is the sole carbon source [117]. *Lactobacillus acidophilus* showed an antioxidant effect in the in vitro fermentation of glucomannan [118]. Co-cultivation of *Lactobacillus* and *Akkermansia* species shows that *Lactobacillus rhamnosus* GG stimulates the growth of *Akkermansia* [119]. 

In summary, *Bifidobacterium* and *Lactobacillus* as common probiotic strains are able to degrade and utilize DOPs and produce SCFAs, inhibit the growth of harmful bacteria such as *Escherichia coli*, and inhibit the growth of *Akkermansia*.

(3) *Akkermansia is* a potential probiotic that is unable to break down glucomannan but can utilize monosaccharides from the breakdown of mucin and glucomannan and break down mucin O-glycans, promoting the growth of mucin-associated bacteria such as butyrate-producing *clostridia.* In humans, many studies have shown a negative correlation between the abundance of *Akkermansia* and overweight, obesity, untreated type 2 diabetes, or hypertension [120,121,122,123,124]. A recent clinical trial in overweight or obese volunteers with insulin resistance showed that oral intake of *Akkermansia* is safe and improves insulin resistance and obesity in the intervention groups [125]. As a potential probiotic, *Akkermansia* utilizes strategies such as the secretion of glycosidases, proteases, sulfatases, and sialidases to degrade mucin [126] or gastrointestinal mucins [127], thereby promoting host mucin secretion and playing an important role in mucosal homeostasis. *Akkermansia* utilizes sialidases and fucosidases to degrade mucin O-glycans and is involved in the cross-feeding of mucin-associated bacteria, e.g., by promoting butyrate secretion from *Clostridia* during co-cultivation [128]. Its digestion pattern is shown in Figure 2. *Akkermansia* itself can ferment mucin to produce acetate and propionate [129]. *Akkermansia biwaensis* sp. nov can utilize mucin as the sole carbon and nitrogen source as well as D-glucose, D-mannitol, lactose, and D-mannose [130].

## 5. Conclusions and Perspectives

**Figure 3:**
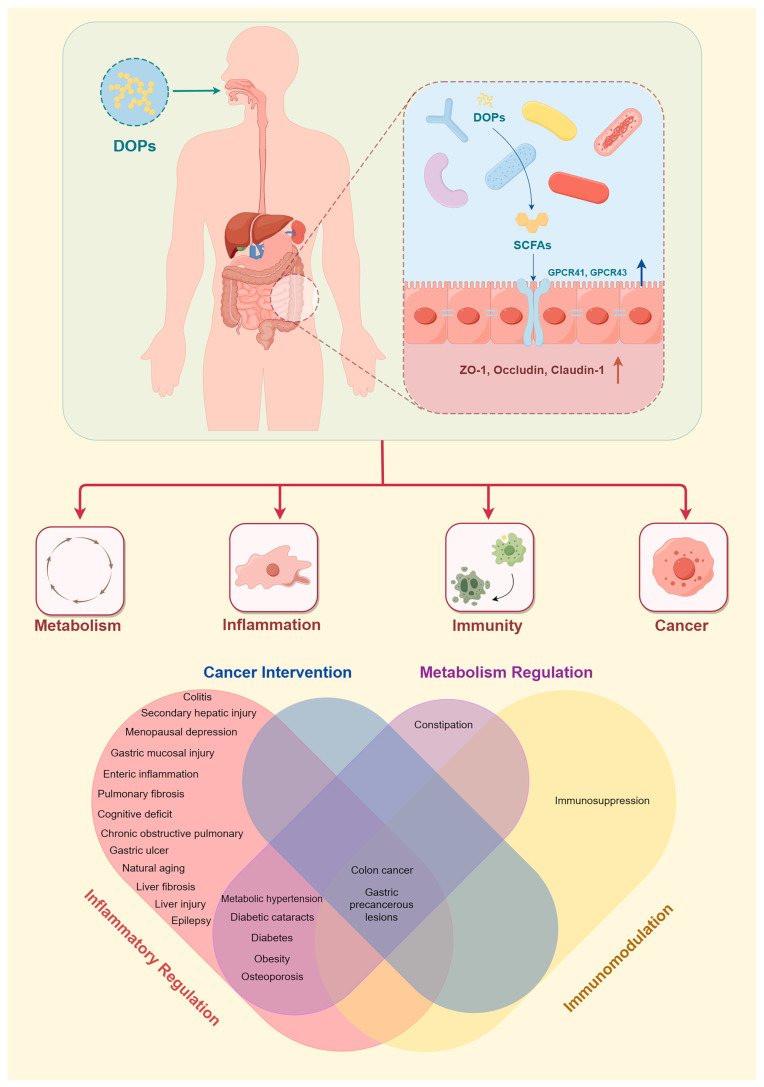
The main mode of action and biological activities of DOPs in health.

Increasing evidence suggests that the gut microbiota plays a crucial role in metabolism, immunity, inflammation, and cancer processes. The metabolites and endotoxins released by the gut microbiota can influence overall health through systemic effects via the bloodstream. DOPs have been shown to influence the gut microbiota, such as the abundance of *Lactobacillus*, *Bifidobacterium*, *Akkermansia*, *Bacteroides*, and *Prevotella*, and provide different benefits to the host due to structural differences. The dietary intake of DOPs has been shown to improve the composition of the gut microbiome and offers new intervention strategies for metabolic diseases such as obesity and type 2 diabetes as well as inflammatory diseases such as chronic obstructive pulmonary disease and colitis. Compared to drug therapy, intervention with DOPs is not specific and has a longer intervention duration. However, as a prebiotic, it has similar positive effects on inflammation modulation to inulin [6] and shows better effects on urea and amino acid metabolism and intestinal barrier protection than konjac glucomannan [10,15]. DOPs show excellent safety and health benefits as a potential prebiotic. The main mode of action of DOPs and the directions of potential treatment of diseases corresponding to the above activities are summarized in Figure 3.

Currently, the relationship between DOPs and the gut microbiota is not fully understood and a clear relationship between potential prebiotics and probiotics is lacking. In the future, the growth-promoting effect of DOPs on probiotics needs to be verified. There is a lack of high-quality clinical trials in humans with chronic diseases such as obesity, type 2 diabetes, hyperlipidemia, and chronic obstructive pulmonary disease considering DOPs as potential prebiotics in dietary interventions. In cancer processes and immune activity, DOPs need to be further investigated as adjuvant therapies or dietary interventions in animal studies. In addition, it is important to consider the structure, dosage, and timing of intervention of DOPs and to increase the yield of DOPs when used in the food industry as a prebiotic. This will facilitate the development of functional foods and health products to improve human health.
